# The struggle to stay physically active—A qualitative study exploring experiences of individuals with persistent plantar fasciopathy

**DOI:** 10.1186/s13047-023-00620-4

**Published:** 2023-04-15

**Authors:** Marianne Mørk, Helene Lundgaard Soberg, Aasne Fenne Hoksrud, Marte Heide, Karen Synne Groven

**Affiliations:** 1 Department of Physical Medicine and Rehabilitation, Oslo University Hospital, Postboks 4956, 0242 Oslo, Nydalen Norway; 2 Institute of Clinical Medicine, Faculty of Medicine, University of Oslo, Postboks 1078 Blindern, 0316 Oslo, Norway; 3 Research and Communication Unit for Musculoskeletal Health (FORMI), Division of Clinical Neuroscience, Oslo University Hospital, Postboks 4956, Nydalen 0242 Oslo, Norway; 4 Department of Rehabilitation Sciences and Health Technology, Faculty of Health Sciences, Oslo Metropolitan University, Postboks 4, St. Olavs plass, 0130 Oslo, Norway; 5 Norwegian Olympic and Paralympics committee and confederation of sports, Postboks 5000, 0840 Oslo, Norway; 6 VID, Specialized University, Diakonveien 12-18, 0370 Oslo, Norway

**Keywords:** Plantar fasciopathy, Qualitative study, Phenomenological framework, Thematic analysis, Psychosocial issues, Semi-structured interviews

## Abstract

**Background:**

Plantar fasciopathy is the most common cause of heel pain, and is associated with decreased physical activity level and quality of life. There has been limited research on the experiences of patients with plantar fasciopathy. This study seeks to gain more in-depth understanding and knowledge by exploring the lived experiences of people with persistent plantar fasciopathy.

**Methods:**

We included 15 participants with longstanding plantar fasciopathy. Face-to-face, semi-structured interviews were audio recorded, transcribed verbatim and analysed using Braun and Clark’s reflexive thematic analysis. We used an inductive approach led by a phenomenological theoretical framework.

**Results:**

We identified three core themes and ten sub-themes. The first theme was ‘Struggling to stay active’ with sub-themes ‘Struggling with pain and how to adjust it’, ‘ Finding alternative activities’ and ‘Longing for the experience of walking’. The second main theme was ‘Emotional challenges’ with the sub-themes ‘Feelings of frustration and self-blame’ and ‘Worries of weight gain and related consequences’. The third main theme was ‘Relations to others’ with the sub-themes ‘Participation in family and social life’, ‘ Visible in new ways’, ‘ Striving to avoid sick leave’ and ‘Bothering others’.

**Conclusions:**

Participants revealed how their heel pain led to inactivity and emotional and social challenges. Pain when walking and fear of aggravating it dominated the participants’ lives. They emphasised the importance of finding alternative ways to stay active and avoiding sick leave. Treatment should focus on holistic and individually tailored approaches.

## Background

Plantar fasciopathy (PF) is the most common cause of plantar heel pain affecting both sedentary and athletic individuals [1]. Among adults over 50 years of age, the prevalence is found to be 9.6% [2]. The diagnosis of PF is primarily based on clinical assessment and medical history [3]. Patients with PF report pain located in the medial tubercle of the calcaneus, often worse in the first steps after rest or prolonged weight-bearing activities [4, 5]. Histologically, PF is described as a degenerative process [6]. The prognosis for PF is uncertain. Hansen et al. followed 174 patients with PF who all received treatment (most frequently treated with corticosteroid injections and insoles). Their results showed that the risk of still having symptoms of PF 15 years following onset was 44% [7].

A wide variety of treatment options are available for patients with PF, although scientific evidence for their effectiveness is unclear. The latest best practice guide from 2021 recommended a stepped approach with stretching, foot taping and educational intervention as the initial intervention for all PF patients [8]. Previous research suggests the management of patients with PF using a biopsychosocial approach rather than a biomedical model [9, 10]. The biopsychosocial model was developed by Engel in the 1980s, and emphasises the interconnection between biological, psychological and social factors when managing patients [11, 12]. This model has some challanging aspects. According to Stilwell and Harman, the boundaries between the three factors are artificial and the model takes a fragmented approach to pain experienced by patients.They suggest a new conceptualization of pain that incorporates the framework of phenomenological research [13].

Individuals suffering from PF report decreased health-related quality of life in comparison with the general population [14]. Symptoms of depression, anxiety and stress are significantly associated with suffering from PF, as are kinesiophobia and catastrophising [15, 16].

Although the negative psychosocial impact associated with PF is well known, there is a lack of qualitative research concerning this patient group. To our knowledge, only one qualitative study exploring experiences of patients with PF has been published. Cotchett et al. found eight themes including perceptions of plantar heel pain, impact of self, dealing with plantar heel pain, source of information, patients’ needs, patients’ unmet needs, advice to others and interests in online education. They concluded that PF negatively affects quality of life and that patients’ needs were often unmet [17].

With an emphasis on including psychosocial aspects along with the biomedical approach in treating patients with PF, the present study seeks to bridge a gap in the literature and contribute with in-depth insight into the bodily experiences of individuals with PF.

The aim of this study is to expand the knowledge base concerning individuals’ every-day experiences living with long-term PF by addressing the following question:

*“What are the experiences, thoughts and concerns of patients with persistent plantar fasciopathy, and how do they cope with everyday life?”*

## Material and methods

### Design

We applied a qualitative approach to achieve insight into patients’ experiences and address our research question. An explorative, descriptive design was used with individual face-to-face interviews. We developed a semi-structured interview guide (Table 1) based on the researchers’ clinical experiences, relevant literature reviews and discussion with a user panel at Oslo University Hospital (OUH). An individual with foot pain also participated in writing up the results and the discussion.

**Table 1:** Topics in the semi-structured interview

1. Background
⁃ Please start by telling us about yourself and the reason why you came to our department
2. Debut of heel pain
⁃ When and how did you first notice that your heel was bothersome? Special episodes?
3. Heel pain development and experiences
⁃ How has the heel pain developed?
⁃ How do you experience the heel pain in your everyday life?
⁃ How do you experience the heel pain compared to other previous pain?
⁃ If you were to picture the heel pain, what do you see?
⁃ If you did not experience this pain, how would your life have been different?
⁃ If you were to describe your heel pain to somebody who has not experienced such pain, what would you say?
4. Cause of the heel pain
⁃ What do you believe caused your heel pain?
5. Coping
⁃ How do you think you have coped with your heel pain?
⁃ What do you consider the most challenging part of living with heel pain?
⁃ Do you avoid anything because of the pain? Examples?
⁃ What have you tried to do to decrease your foot pain?
⁃ What advice would you give to a friend with the same heel pain?
⁃ What challenges do you think you have overcome in spite of your heel pain?
6. The future
⁃ What are your thoughts about your heel pain in the future?

### Setting, recruitment and participants

We included patients with clinical PF from the department of Physical Medicine and Rehabilitation at Oslo University Hospital, Norway (OUH). Patients were eligible after a clinical examination by experienced, physicians and specialists in physical medicine. The study was performed in connection with a randomized controlled trial (RCT) with the title: “The effect of radial extracorporeal shock wave therapy (rESWT), sham rESWT, standard exercise program or usual care for patients with plantar fasciopathy [18] (ClinicalTrials.gov NCT03472989). Inclusion criteria were; age between 18 and 70 years, pain intensity of 3 or more on a numeric rating scale (NRS), pain duration of over 3 months, pain localized in the proximal insertion of the plantar fascia on the medial tuberosity and tenderness to palpation to the painful area. Exlusion criteria were; treatment with radial shock wave therapy for the least 3 months, sponyloarthropathy or rheumatoid arthritis, plantar fibromatosis, tarsal tunnel syndrome, polyneuropathy, previous surgery with remaining osteosynthesis material in the foot or ankle and contraindications for radial shock wave therapy (use of anticoagulant drugs, pregnancy, bleeding disorders, epilepsy or pacemaker).

Participants who fulfilled our inclusion criteria were given oral and written information about the study. The assessing physicians asked patients to participate in the qualitative study with interviews. An appointment for the interview was scheduled with the first author, a female physiotherapist and PhD student with extensive experience assessing and treating foot patients. The participants were aware of the interviewers’ profession, but there was no therapeutic relationship between the two parties prior to the interviews.

In order to capture a range of characteristics, we used a purposeful selection method based on gender, age, body mass index (BMI), work status, duration of symptoms and pain intensity. The concept of “information power” was used to guide sample size [19], and we used consolidated criteria for reporting qualitative research (COREQ) to ensure quality [20].

All patients who were asked to participate in the current qualitative study, agreed to participate. Fifteen interviews were completed in the period between 11.06.20 and 27.8.21 with eleven females and four males aged between 31 and 65 years. Participant characteristics are presented in Table 2. The interviews lasted between 39–62 min (mean 51 min). Notes while interviewing and immediate reactions afterwards were written down by the interviewer.

**Table 2:** Participant characteristics with fictitious names

Names	Age	Work status	Physical activity level	BMI	Duration symptoms	NRS
Anne	57	100% work	walking/ biking ≥ 4 h/w	27.4	12–24 months	6
Beate	33	unemployed	walking/ biking ≥ 4 h/w	26.8	> 24 months	5
Kristin	48	100% work	walking/ biking ≥ 4 h/w	30.7	6–12- months	7
Elisabeth	46	100% work	recreational sport ≥ 4 h/w	24.3	3–6 months	5
Fredrik	51	100% work	walking/ biking ≥ 4 h/w	31.9	3–6 months	7
Hanna	54	70% sick leave	walking/ biking ≥ 4 h/w	27.5	3–6 months	5
Julia	65	50% disabled	walking/ biking ≥ 4 h/w	26.2	6–12 months	10
Kine	43	50% sick leave	walking/ biking ≥ 4 h/w	25.2	12–24 months	9
Mary	47	100% disabled	walking/ biking ≥ 4 h/w	33,4	12–24 months	10
Naomi	41	100% work	walking/ biking ≥ 4 h/w	27	3–6 months	9
Oda	31	100% work	walking/ biking ≥ 4 h/w	27.4	> 24 months	7
Pernille	38	20% sick leave	walking/ biking ≥ 4 h/w	33.5	12–24 months	7
Robert	54	100% work	exercise/competition	27	6–12 months	8
Thomas	58	100% work	walking/ biking ≥ 4 h/w	26.8	3–6 months	7
Victor	49	100% work	walking/ biking ≥ 4 h/w	33.1	12–24 months	8

Age in years, h/w: hours per week, BMI: Body mass index, NRS: Numeric rating scale, pain in activity, sick leave: employees abcent from work due to sickness, disabled: work disability due to sickness, unemployed: able to work, but have no paid job

### Data collection

The first author conducted all interviews in a conversation room at the department at OUH. The location was chosen for its convenience. The interviewer wore casual clothing to make the atmosphere feel less like a hospital. Two pilot interviews were conducted by the first and last author, the latter a physiotherapist and professor, having extensive experience with qualitative research.

The thematic interview guide was used as a framework. Unexpected and interesting themes were explored further when they came up. We focused on the participants’ experiences living with PF and encouraged them to elaborate on them in their own words.

### Analysis

The interviews were audiotaped and transcribed verbatim by the first author. The first author and two co-writers (second and last author) conducted thematic analysis (TA) in line with Braun and Clark’s recommendations. In 2006, Braun and Clark described a method within TA for analysing and interpreting patterns across the data by using coding to develop themes [21]. Their data analysis emphasizes the importance of reflexivity, theoretical knowingness and transparency [22]. We conducted a collaborative and reflexive process in which we followed the six phases introduced by Braun and Clarke [23]. An inductive approach was used in the analysis [24]. Inductive analysis is described as being driven by the data, in the sense that theoretical framework is included as an interpretive lens in the final stage of analysis [25].

The first author familiarized herself with the dataset by listening to the recordings, transcribing all the interviews and reading the entire dataset. Initial codes were generated systematically and comprehensively throughout the whole dataset to identify codes, which could represent relevant patterns with reference to our research question. The codes were collated and relevant data segments were included under each code. Clusters of codes which were relevant to the research question were identified and initial themes were generated through collaboration between the first, second and last-authors. Tentative themes were identified, reviewed and checked if they presented relevant and recurring patterns related to the codes and the whole dataset. We refined and demarcated the themes multiple times throughout the analysis. We endeavoured to create informative and concise thematic names. All authors and a user participant were involved in the last phase of writing the article with the aim of giving the reader a comprehensive and trustworthy account that reflected the whole dataset and which addressed the research question.

### Theoretical framework

After completing the first stages of analysis of our data, we interpreted the findings through a body phenomenological perspective inspired by the ideas of Drew Leder, and inspired by Maurice Merleau-Ponty as well as Kristin Zeiler and Havi Carel [26–30]. In line with our research question, this framework is relevant when exploring experiences of heel pain as expressed by the participants from a subjective lived bodily perspective.

According to philosopher and medical doctor Drew Leder, long standing pain entails a distressed body which involves being “*stretched apart* from our customary lives and from one another” ([27], p.1). Leder characterizes pain as a source of frustration and struggle, which, again, enhances pain [27]. Leder’s ideas build on Merleau-Ponty’s body phenomenology, focusing on human perception as our primary experience of the world. The lived body is, according to Merleau-Ponty, the experience from a first-person perspective [28]. Leder emphasises the integration of mind and materiality related to the embodiment [27]. A contrasting perspective is that of the corporal body, which is the experience from a third person perspective. Merleau-Ponty criticises this intellectualist understanding of the body as distinct from the mind, and claims that people being in-the-world as body-subjects creates meaning in the world [28].

Bodily dis-appearance, as described by Leder, is present when the subject can carry out a task, for example walking, without having to explicitly focus on how to move their body; it is experienced when the mind and body act harmoniously. The body can dis-appear, or be “absent”, and attention is then drawn towards our surroundings [26]. The dis-appearance of the body can be disrupted if, for example, a person starts feeling pain in their legs. The attention shifts towards the painful legs and the body is no longer “taken-for-granted.” The body dys-appears, in the sense that it appears to us as “ill” or “bad” and comes to the forefront of our attention [26]. The previous bodily certainty will now be questioned. In line with these ideas, Carel introduces three components of bodily doubt: the body becomes thematised as problematic, a pain-free and functional body can no longer be taken for granted and, lastly, concern, fear and anxiety develop [31]. Leder presents the painful body part as an “alien” or how the person in pain may feel alienated from meaningful things or the people around them [26].

Building on Leder’s dis-and dys-appearance, Zeiler introduces the phrase eu-appearance: “in these cases the body stands forth, to the subject, as well, easy or good” ([29], p.338). Eu-appearance may be experienced when walking, when feeling a nice breeze while enjoying the movement and work of the body.

The shift in bodily awareness does not occur in isolation. Leder emphasises the intersubjectivity concerning dys-appearance, he writes: “My awareness of my body is a profoundly social thing, arising out of experiences of the corporeality of other people and of their gaze back upon me” ([26], p. 92).

## Results

Our analysis generated three main themes and ten subthemes. The first theme was:*‘*Struggling to stay active’ with three sub themes:‘Struggling with pain and how to adjust it’, ‘ Finding alternative activities’ and ‘Longing for the experience of walking.’ The second core theme was; ‘Emotional challenges’ with the two sub themes: ‘Feelings of frustration and self-blame’ and ‘Worries of weight gain and related consequences.’ The third main theme was: ‘Relations to others’ with the four sub-themes:’Participation in family and social lives’, ‘ Visible in new ways’, ‘ Striving to avoid sick leave’ and ‘Bothering others.’ We will present our findings thematically.

### Theme 1. Struggling to stay active

Participants experienced intense pain during weight-bearing activities and considered being unable to walk and run as major problems restricting them in their everyday lives.

#### Sub-theme 1. Struggling with pain and how to adjust it

Participants described how they struggled to plan their days, for example by postponing activities involving walking until the evening, in order to manage everyday life. Fear of intensifying pain increasingly came to predominate their priorities and participants spoke of daily negotiations of whether to push themselves and be active or to stay at home. As Julia put it:

*“Yes, I avoid going out. Because I know that when I get home after walking for a bit, I will be in a lot of pain and then it's ruined. When I go shopping, I do it late in the day because when I get home it’s evening and then I go to bed. I'm not afraid, or actually, in a way, I am. Because I know that when I get home I will be in a lot of pain and I won't be able to do anything else. I have friends who have cars, they drive me sometimes, but I don't go if I have to take the bus, just the thought that I have to walk from there to there, no, then I stay at home, but what should I do?”*

In a similar vein, Robert, who had previously been active on a regular basis, noted that he stopped exercising because of the pain:

*“But it hurt when I ran, really hurt. It was probably a month when I stopped doing everything, no exercise at all.”*

#### Sub-theme 2. Finding alternative activities

In the first period of foot pain, many participants tried resting to alleviate it. However, many did not feel that avoiding activity was helpful, and that inactivity had a negative impact on their health. Those who managed to find alternative non-weight-bearing activities, considered them to be important in handling their heel pain*.* Hanna used biking as a substitute for walking:

*“I spent the summer cycling. Luckily, it has been summer, otherwise I would have gone crazy a long time ago because I don't have the patience to not be able to walk. I've never been that sporty, but I've always liked walking. And that's what I can't do. That bike saved my mental health.”*

In a similar vein, Elisabeth challenged herself with learning a new sport in order to stay physically active:

*“But I've been quite good at finding alternatives. So, for example, I've taken a kayaking course, so now I've sort of become a kayaker.”*

#### Sub-theme 3. Longing for the experience of walking

Many participants missed being as physically active as they had been prior to the heel pain. The positive feelings experienced while walking or running were expressed by the participants with the use of different metaphors. Pernille explained how walking felt like meditation for her:

*“Walking as exercise is really the best. It's the best for me mentally too. I actually love to walk. So, I think that walking is the best because then you are physically active at the same time as you are meditating inside, in a way.”*

In a similar vein, Hanna felt walking was good for her mental health:

*“You get to clear your head and thoughts when you walk.”*

Many participants expressed how physical activity sparked a good cycle with more energy and a better mood. As Naomi put it:

*“Because I really love going for walks, I really love being outside.... I don't know how to explain it, getting fresh air. I feel like I get so much energy, and I feel like I've been reborn and I'm ready to do lots of good things.... I get stiff in my pelvis and back if I don't walk. And then I comfort eat a lot too.”*

Elisabeth explained how she felt while out jogging, prior to the heel pain:

*“To be able to go jogging and to be able to smell the spring and things like that. It was just such a completely wonderful feeling of freedom.”*

### Theme 2. Emotional challenges

Experiences of heel pain and not being able to walk and be active in the ways they had been was immensely challenging, and participants spoke of how anger, worry, sadness, impatience and frustration increasingly predominated their everyday lives.

#### Sub-theme 1. Feelings of frustration and self-blame

Having to change their expectations and plans due to heel pain was described as a source of irritation and feelings of hopelessness by many participants. Kine felt that even though she had tried to do the right things to get rid of the heel pain, it persisted. Furthermore, she became self-blaming and stressed about others’ expectation. She revealed:

*“What exactly is the problem, why can't I fix this? I always, like, wear trainers and have always been good at being careful, I don't wear high heels, I just don't understand why …no, it’s more that I'm more frustrated with – and can't I just do the right things so I can get well? What am I doing wrong now? Something is expected of you all the time, the children and my husband ask; are you okay now? It's better now, right? You know, I would like to answer, YES now I'm done (laughs) it’s not bothering me anymore, but it is. Oh, extremely stressed, very stressed, I feel that now I'm not achieving, now I should have delivered.”*

Others expressed how inactivity also triggered self-blame, like Anne:

*“Because you are so dependent on the feet to transport you. I have noticed that it is easier to take a car to places I would normally walk or cycle to. And that makes you get lazy and then you get annoyed with yourself because you are lazy*.”

Anne also explained that she felt resigned at times and gave in to the pain:

*“You simply become a bit defensive, whether it effects the brain, I don't know. But at least it does something to you mentally that you give up a little. I also think that no, no, that was how it should be, and you don't deal with it like you might have done otherwise.”*

#### Sub-theme 2. Worries of weight gain and related consequences

Many participants expressed their worries about the possible consequences of inactivity over longer periods of time. Kristin did not manage to stay active; she felt bloated and was concerned:

*“Yes, I can't walk, like, from February until May, I've barely moved, I've just been sitting. I feel like my body is swelling up, I feel sluggish, I'm also worried about my health, you know? I'm a little overweight. I borrowed a rowing machine from some friends. But everything hurt, so I haven't managed to get my heart rate up. At least to get my heart rate up and be a bit active, then I get a little worried about my health, then I think that's my menopause, so now it's like everything is falling apart.”*

Naomi described how she had previously used exercise to control her body weight. After gaining weight, she felt dissatisfied and unhappy with herself:

*“I've put on weight too, a lot and that's the worst thing to me. I was a bit overweight for a while, so I tried to work on it and lose weight. For many years I have been very active, been very good at eating and exercising a lot and running with my dog for 1 hour, going to the gym for 1 hour after that. So, I've been very strong and worked very hard, so it should be the body you feel happy with. It's even more depressing when I'm not happy with myself, then I don't have so much to give to other people.”*

Elisabeth explained how she felt dependent on physical activity in order to maintain both her fitness and mood and that she became distressed by the fear of being unable to continue exercising:

*“No, like, I depend on being able to exercise regularly in order to somehow maintain my mood, fitness and weight. If I stop doing it, I'm afraid that, yeah, I think it will go downhill, that is, it will go downhill in all those areas. I have very few options these days, I feel. A bit high risk and a lot of uncertainty. Yeah, last week those who had been to the gym between then and then had to quarantine, but it didn't happen after all. Then I actually panicked a bit. Then life gets very boring and now I have sort of been on a roll where I have exercised regularly. And if, like, it doesn't work out, I'm afraid that it will be hard to get back into it again. No, then I get a bit worried. Like, it sounds so trivial.”*

Robert reported worries about serious illnesses due to inactivity and expressed a need to recover quickly from PF in order to take care of his family**:**

*“I've heard that a lot of people who don't exercise get high blood pressure, there are lots of people I hear about who die … yeah, I'm a bit scared. My father died from cardiac arrest. I have to be fit, I don't like being sick and staying home... I’m never sick, not on sick leave. I'm healthy … no, it's sad, that's why I'm going to the doctor and have to hurry up and get well. Because some say one year or two years. TWO YEARS.”*

### Theme 3. Relations to others

Participants explained how the heel pain changed and limited their participation in social activities with family, friends and colleagues. Furthermore, some spoke of how the pain and its related consequences changed their relationships with others and how the pain influenced their work.

#### Sub-theme 1. Participation in family- and social life

Deprived of the ability to participate in social activities with family and friends as before was described as challenging, giving rise to feelings of guilt, frustration and decreased social participation. As Naomi described it**:**

*“So, I just have to tell my son, he says: Mum, can't you run with me? Because he is used to his mum running with him and going for long walks and us having fun in the forest and things like that, but we don't do that anymore. He asks me all the time and I have to keep telling him I'm in so much pain, I'm in so much pain.”*

Elisabeth had a history with knee pain. She had recovered and was looking forward to running again, but the heel pain made it impossible:

*“I was very annoyed at home because I had been looking forward to being able to start jogging again. Then I was in a really bad mood when I couldn't jog because I had thought that I could jog with my daughter.”*

In a similar vein, Anne explained how her social life had become restricted because she could not participate in physical activities with her friends:

*“We were a whole group of friends who were going to go to the mountains, but I couldn't. I walked a bit, then I had to turn back. And it's things like that that I find tiresome. Not being able to walk, because then it hurt so much that it was very uncomfortable to walk. A little less social, there are certain things I haven't been too keen on joining. It is this type of physical activity because I know it has been painful for me.”*

#### Sub-theme 2. Visible in new ways

Heel pain caused gait impairments such as limping, reduced pace or different weight-bearing patterns e.g., walking on their toes or outside of the foot. These restrictions were impossible to hide and participants spoke of becoming visible in new and somewhat embarrassing ways. Kristin explained it as follows**:**

*“I also think it's so embarrassing at work because it can look like I'm limping, and it's only at the start, and it can happen that I walk across the school yard, but by then I've sort of got the hang of it so that I'm able to walk almost apparently without being affected by pain. I feel so stupid.”*

Similarly, Kine disliked the attention she got when she used crutches:

*“It annoys me that people question me when they see that I'm limping. I say, but it's fine. At that time I had to walk with crutches because it was so painful. Oh, so it was, like, that if I saw someone I knew, I tried to wait until they had passed to avoid them.”*

Naomi compared herself to a turtle to emphasise how depressed she felt when she was unable to keep up with friends and colleagues:

*“I walk very slowly, I take a very long time, I have the speed of a turtle. I see people coming and I can't even walk. Very depressing, sometimes when my colleagues stand there and greet me, I say now you just have to go, you know?”*

#### Sub-theme 3. Striving to avoid sick leave

The majority of the participants stayed in work and considered this to be crucial for coping with the heel pain. For many, this required a lot of effort and they took pride in staying in work. Fredrik explained that sick leave was not an option for him:

*“As long as I have enough to do at work, I don't really think about it too much, so it's fine. I'm at work every day, so, I have to say, I handle it pretty well. I could have laid down or called in sick and said that this isn’t going to work. It's not coping, is it? I’ve decided that I would have to be quite sick to not make it to work, so to speak.”*

Participants explained that being preoccupied with work reduced their focus on the heel pain and described finding meaning in feeling useful and a part of a community. Julia put it like this:

*“I think it's very good to be at work. I enjoy being at work and I don't like being at home, so it’s, like, psychological help. And what can you do at home? Then you only think about pain, when you can't be very active. I think that going to work is the best for me, then I don't think. Then I am with the people I love. We have such a great time, fantastic. Yeah, that I’m able to go to work, yeah, I’m proud of that”.*

Ignoring the pain and striving to stay in work was described by many participants. However, as the pain increased, remained for longer periods and affected their sleep, some felt it was impossible to continue working*.* Hanna told of her experience:

*“I went a long time without mentioning it, I don't really know why. If you just pretend things aren't there, then they go away. I think it has gotten better, because I get relief when I'm on sick leave? It worked very badly to just keep stomping around and pretending that there wasn’t anything to make allowances for. No, it really hurt during the evening and into the night at its worst.”*

Some participants were on sick leave, especially those with occupations involving a lot of walking or standing. Being on sick leave felt psychologically challenging, as Victor revealed:

*“And it's tough. I don't think I'm being kind to myself when I go home every day and don't manage to go for a walk and sit alone. Ugh, to look people in the eye, I feel it’s negative that I'm on sick leave. Patience, I wish you had, like, a pink pill that you take it and then you’re free of the problem. I know I'm impatient, but there's a lot behind being on sick leave, you feel like you're a burden, being on sick leave they have to have substitutes and things like that ... Yeah, also I’m impatient now I'm also tired and have a bit of a shorter fuse because I sleep a little badly.”*

#### Sub-theme 4. Bothering others 

When explaining how they felt they handled the heel pain, many replied that keeping the pain to themselves and not bothering others with their suffering was important for them, as Oda put it**:**

*“So, I try not to let my pain affect anyone else. Of course, you have to talk about it with someone else because it's tiring, but you don't, like, complain about it all the time. I’m not really a complainer.”*

In a similar way, Hanna talked about how she was uncomfortable with what she described as whining. Asking others for help was also difficult for her:

*“No, it is not comfortable being a complainer. You also do everything anyway because it takes less time to do things yourself than to explain to people, right? I'm not the patient type. Obviously, it didn’t make the burden any lighter that I walked terribly much anyways.”*

## Discussion

In this study, we aimed to explore the experiences of individuals with longstanding PF. To address our research question, we will discuss our findings with reference to the body from a phenomenological theoretical perspective, supplemented with previous research.

A major finding in the study was that participants regarded pain during and after walking as the most problematic aspect of living with PF. They experienced fear of pain and uncertainty as they struggled to find a balance between challenging their pain and trying not to aggravate it. The participants expressed how their foot pain became the focus of their attention, which can be interpreted following Drew Leder’s notion of the dys-appearing body. They could not take their body for granted anymore, and experienced their body as an obstacle in everyday life. Our participants felt uncertainty and were constantly negotiating with themselves about weather to participate in painful activities or not. These doubts led them to continuously monitor their pain, and dominated their decisions regarding avoiding painful physical activities, limiting their freedom to move. These experiences are consonant with the ideas of Carel [31], who suggests that consciously planning everyday activities leads to bodily uncertainty and limitations.

Our study highlights the participants’ challenges with having to adjust their physical activity. While some expressed negative emotions associated with becoming significantly less active, others elaborated on the importance of finding alternatives to weight-bearing activities when coping with PF. Participants felt that swimming, biking, rowing and strengthening exercises were all feasible activities that contributed to positive experiences. According to Leder, individuals dealing with longstanding pain will alternate between being productive and in control and out of control when pain dominates, which will in tern determine their feelings and actions [27].

In line with our results, Cotchett et al. found that PF had a negative impact, in particular in limiting the ability to walk, run and stand for long periods of time [17]. In a recent mixed-methods systematic review on psychological factors associated with foot and ankle pain, the same researcher found that experiencing foot pain influenced emotions and function, which they associated with pain-related fear [10]. These results support the findings in our study concerning fear of foot pain and uncertainty about how to handle it.

Participants in our study emphasised the importance of walking prior to being diagnosed with PF. Elaborating on these prior experiences, they used phrases like ventilation for the mind. Many participants experienced better mood, more energy and the feeling of happiness when walking. Prevented from walking as they had before, participants described a longing for the experiences of eu-appearance when walking, a bodily awareness in which mind and body work harmoniously together. According to Zeiler, “The body can eu-appear to me as something that I am aware of as positive without this being an explicit focus for my attention and without its disrupting my way of being or acting” ([29], p.341).

Participants in our study described how their heel pain led to a limping gait, the need for crutches or being unable to maintain the same pace as others which made them feel embarrassed and uncomfortable. The body became thematised as a problem disrupting communication with others, giving rise to social dys-appearance [26]. As far as we are aware, these findings are the first to touch upon the theme of how a limping gait or being unable to walk at a normal pace are experienced by individuals with heel pain.

In line with our findings, previous research has highlighted the negative impacts of being unable to walk and stay physically active. Cotchett et al. documented reduced ability to walk, negative emotions and social isolation as impacts of plantar heel pain [17]. Turner et al. described how individuals with Achilles tendinopathy experienced a restricted ability to walk and run leading to frustration and annoyance [32].

The participants in our study experienced their heel pain at present as severe and overriding, giving rise to feelings of powerlessness regarding how to influence or speed up the healing process. Leder describes the temporality of pain with the presence of the pain as endless and projective impacting hopes, dreams and aspirations of the future. He argues that pain can create a feeling of imprisonment that limits freedom of movement and leads to disconnection with the surrounding world. Although the future may appear uncertain for people with pain, it may also present possibilities and the search for a pain-free future can represent hope. According to Leder “… the chronic pain is truly the time itself (khronos), unless and until the sufferer can make peace with this three-faced god of past, present and future” ([27], p.32). Our results show that the participants experienced frustration and psychological distress due to their present pain and being unable to stay as physically active as they used to as well as, sadness for what they missed out on, but, at the same time, many had hope for the future, albeit with some uncertainty.

In line with our findings, Turner et al. documented similar results of frustration, impatience and feelings of hopelessness when exploring the experiences of people living with Achilles tendinopathy [32]. In a similar vein, Cotchett et al. found comparable results in their qualitative study with patients with PF, describing the frustration with the metaphor of a mouse stuck on a wheel [17].

According to our findings, participants experienced that heel pain limited their participation in social activities. Being unable to join friends and family in physical activities like they used to, such as walks in the woods or hiking in the mountains, triggered feelings of loneliness and distanced them from those closest to them. They experienced guilt for not being able to contribute to family life as they had before, and increasing the burden on others. Leder argues that chronic pain may dis-place and pull the sufferer away from their usual place in the world and from their routines and their fellows [27]. According to Svenaeus [33], chronic pain alienates the sufferer both from themselves and from the outside world. These ideas tally with the feelings of loneliness and guilt expressed by the participants in our study. Participants felt separated from their families and friends because they could not take part in shared activities anymore.

The negative impacts on social life caused by living with foot pain have also been demonstrated in previous qualitative research with patients suffering from PF [17], and among those with foot pain due to rheumatoid arthritis and Achilles tendinopathy [34–36].

According to our findings, concerns regarding being overweight, in the sense that their BMI was classified as overweight or obese (BMI range from 24–33), predominanted. Participants commonly feared developing lifestyle diseases, but also mental illnesses like depression due to weight gain. Those who had previously emphasised intensive physical activity as a means of preventing overweight, anxiety or depression, worried about their future in terms of how stress and fear would develop. Leder describes being in control and out of control as a paradox of pain ([27], p.36–38). Participants in our study juggled feelings of being in control and of losing control; they often felt overwhelmed, passive and out of control when handling their pain, but, at other times, some felt in control when finding alternative ways to stay active and avoid weight gain. These experiences are reflected in contemporary Western society’s emphasis on physical activity as essential to staying healthy and as an active treatment for different diseases. Health authorities repeatedly emphasise evidence-based advice on the type and frequency of physical activity we should include in our daily routines in order to stay healthy [37]. Participants in our study explained how they used to follow these recommendations by walking, running and engaging in other weight-bearing activities. Being aware of the importance of physical activity, but being hindered by foot pain, seemed to make the individuals in our study feel out of control, which, in turn, gave rise to health-related fear.

Previous research has identified difficulties with weight loss when individuals are unable to be as physically active as usual due to heel pain [17]. However, the findings in our study on participants’ fears of being overweight and developing lifestyle diseases, have, to our knowledge, not been described previously in the foot literature.

Our results highlight the importance of being engaged in meaningful work when living with long-term heel pain. The participants explained how they strove to avoid sick leave. They described the value of working on a daily basis and interacting socially with collegues, both of which helped them to focus on something other than pain. They worried about how they would cope on sick-leave, and feared experiencing isolation, depression and an increased focus on their pain. Some of the participants struggled for a long time at work, trying to ignore their foot pain and not involve their employer or colleagues. When the pain began affecting their sleep, it became impossible to perform their duties at work and they had to go on sick leave. Being on sick leave seemed to entail less heel pain for those with standing or walking jobs, however, participants found it hard to come to terms with both in regards to their own self-respect as well as the fear of losing the respect of others.

Along with the desire to remain in work, participants explained how avoiding involving family, friends, colleagues and employers was the best way to handle the pain. They did not wish to be seen as whiners, and many were uncomfortable asking others for help. Zeiler suggests that lack of social support when in pain contributes to a deepened feeling of social dys-appearance [29]. She argues that the intersubjective dimensions of help are a means of softening the social dys-appearance while being rejected can aggravate the dys-appearance.

According to Leder, social dys-appearance develops when the “gaze of others” is experienced as distanced and objectifying. He also emphasizes the importance of intersubjectivity. “My awareness of my body is a profoundly social thing, arising out of experiences of the corporeality of other people and their gaze directly back upon me” ([26], p.92). Leder’s theories of social dys-appearance are in line with our participants’ experiences of not wanting to involve others in their pain and feelings of isolation. Individuals in pain sometimes isolate themselves from others in order to conceal their pain or avoid being reminded of what they have lost [27].

Some of the participants in our study experienced fear of being disbelieved by others, in particular by their colleagues and employers. Not being met with empathy made the participants hesitant to involve others in handling their pain. According to the philosopher Havi Carel, empathy is the human emotion in greatest shortage. She shares her experience when suffering from a severe disease, saying that the lack of empathy, was what hurt the most [30]. By not involving others, our participants avoided focusing on their painful heel, and the fear of being objectified or not being met with empathy. On the other hand, by not reaching out for support, our participants missed out on empathy, compassion and support from others.

Few participants in our study were on 100% sick leave. Work status is, as far as we can see, not a common factor in descriptive information in quantitative or qualitative studies concerning foot and ankle disorders. Studies demonstrating the negative or positive consequences of sick leave have previously been called for [38]. In a study from 2021, Adams et al. found that employees attending work and suffering from chronic pain experience higher levels of psychosocial stress than those without pain [39]. Although the majority of the participants in our study managed to stay in work and believed it helped them to for cope, they might also experience increased pain and higher level of psychosocial distress which could influence their recovery from heel pain.

As documented in previous studies, patients with PF and other foot conditions report that the pain affects their participation in social life, leading to a feeling of isolation [10, 17]. The importance of being met with empathy and a feeling of being taken seriously by healthcare providers is emphasised by Mallows et al. who performed a qualitative study with patients with Achilles tendinopathy and their perspective on participating in rehabilitation. The participants identified individualised approaches and addressing fears as among the elements that mattered to them [40].

### Strengths and limitations

Some issues highlighted in our study have not been addressed by previous research and therefore expand the knowledge in this field. Furthermore, this new knowledge would not be fully captured by using PROMs. We argue that the new themes addressed in our study also are relevant to other foot populations. The participants in this study were exclusively included from a specialist setting and 73% were women, which could limit the diversity of the population and thereby its transferability. We selected our sample purposely in order to create variation and richness of the data. In our study, the first author collected all the interviews, but the analysis was conducted through dialogue with the co-authors. The interpretation of the qualitative data is influenced by the background and views of the authors, and we have provided a information on the authors and their involvement with the aim of presenting a transparent process.

## Conclusions

Our results demonstrate how heel pain permeated the participants’ lives in a complex and intertwined way. The suffering from persistent PF does not seem to be limited to the foot alone. Participants in our study explained how they struggled on an individual level, but also how their pain affected their relationship with others and their surroundings. Aggravated pain while walking and the efforts to stay physically active predominated the participants’ lives. Furthermore, feelings of frustration, hopelessness, self-blame, dimished social interaction with others and worries of weight gain and its related consequences became daily experiences for participants with persistent PF. Nevertheless, the participants highlighted the importance of finding alternative ways to stay physically active and continue working despite the pain.

### Clinical implications

The clinical implications of these findings point to supplementing a bio-psycho-social perspective with a bodily phenomenological approach acknowledging the lived experiences of pain in order to provide holistic and individually tailored care for individuals with persistent PF. Clinicians should help individuals with PF to find ways to stay in work and remain physically activity, either with load management regarding weight-bearing activities or by finding alternative activities. Furthermore, our results suggest that it is important to show empathy and interest regarding the patients’ concerns about living with longstanding heel pain. Caregivers should seek to understand each unique subjective experience of people living with PF in order to help individuals trapped with stubborn heel pain.

## Data Availability

Anonymized data used in the present study can be made available from the corresponding author on reasonable request.

## Abbreviations

PF: Plantar fasciopathy
PROMs: Patient-reported outcome measures
OUH: Oslo University Hospital
RCT: Randomized controlled trial
rESWT: Radial extracorporeal shock wave therapy
NRS: Numeric rating scale
BMI: Body mass index
COREQ: Criteria for reporting qualitative research
TA: Thematic analysis
